# Pre-operative intraocular pressure does not influence outcome of trabeculectomy surgery: a retrospective cohort study

**DOI:** 10.1186/s12886-015-0007-1

**Published:** 2015-03-05

**Authors:** Nisha Nesaratnam, Nicholas Sarkies, Keith R Martin, Humma Shahid

**Affiliations:** School of Clinical Medicine, University of Cambridge, Hills Road, Cambridge, CB2 0QQ UK; Department of Ophthalmology, Addenbrooke’s Hospital, Cambridge University Hospitals NHS Foundation Trust, Hills Road, Cambridge, UK; John van Geest Centre for Brain Repair and Cambridge NIHR Biomedical Research Centre University of Cambridge, Cambridge, UK

**Keywords:** Pre-operative intraocular pressure, Trabeculectomy

## Abstract

**Background:**

To investigate whether pre-operative intraocular pressure (IOP) predicts outcome of trabeculectomy surgery in patients with primary open angle glaucoma over a 3-year period of follow-up.

**Methods:**

Retrospective cohort study, of a total of 61 patients (80 procedures) who had undergone trabeculectomy surgery after failed medical management at a single centre between 2000 and 2011. Patients were identified through surgical logbooks. A subsequent case note-review identified 61 patients (80 procedures) with primary open angle glaucoma (POAG). The primary outcome was success of trabeculectomy surgery, with failure defined as intraocular pressure (IOP) > 21 mmHg, ≤ 5 mmHg or not reduced by 20% at two consecutive follow-up visits 3-months post-operatively. Qualified success was defined as surgical success with the use of supplemental medical therapy. Secondary outcomes included visual acuity, Humphrey visual field MD, surgical complications and post-operative interventions.

**Results:**

At 3 years, the odds ratio of failure was 0.93 per mmHg pre-operative IOP (95% C.I. 0.83-1.03, p = 0.15 Wald *Χ*^2^ test), and the odds ratio of failure or qualified success was 0.96 (95% C.I. 0.89-1.04, p = 0.35). The incidence of surgical complications showed an odds ratio of 1.02 per mmHg pre-operative IOP (95% C.I. 0.95-1.10, p = 0.55 Wald *Χ*^2^ test). The incidence of post-operative interventions showed an odds ratio of 1.01 per mmHg pre-operative IOP (95% C.I. 0.94-1.09, p = 0.80 Wald *Χ*^2^ test).

**Conclusions:**

Pre-operative IOP does not predict success of trabeculectomy surgery in POAG patients during the first 3 years of follow-up. The incidence of surgical complications and post-operative interventions shows no association with pre-operative IOP.

## Background

Intraocular pressure (IOP) is well recognised as the only modifiable risk factor proven to influence the progression of glaucoma. If medical management is not successful in controlling IOP, surgical intervention may be required. The commonest surgical procedure performed for glaucoma worldwide is trabeculectomy [1,2]. Trabeculectomy is performed for many types of glaucoma, including situations where there is progressive glaucoma despite a relatively low IOP [3]. Long-term follow-up studies of trabeculectomy surgery confirm that it is a successful method of achieving long-term IOP control [4,5]. Although most patients undergoing trabeculectomy surgery have elevated IOP, a significant proportion of patients have never had a documented IOP over 21 mmHg. In general, a lesser percentage reduction in IOP is achieved if the pre-operative IOP is lower, but it is unclear if the pre-operative IOP prior to surgery has any influence on effectiveness of surgery in preventing progression of glaucoma [4]. Previous studies in the literature have only stratified outcomes of trabeculectomy based on categorical definitions of pre-operative IOP, where patients are classified as having normal tension glaucoma when IOP is consistently less than or equal to 21 mmHg, and primary open angle glaucoma when the IOP is consistently greater than 21 mmHg. In the current study, our aim was to determine if the outcome of trabeculectomy surgery for primary open angle glaucoma is influenced by pre-operative IOP when IOP is considered as a continuous variable.

## Methods

This study was retrospective and adhered to the Declaration of Helsinki. It was deemed by the Research and Development Department at Cambridge University Hospitals NHS Foundation Trust to not require ethical review by a Research Ethics Committee.

### Eligibility criteria

Patients who had undergone primary trabeculectomy surgery between 2000 and 2011 at Addenbrooke’s Hospital, Cambridge, were identified through surgical logbooks and a subsequent case note-review. Inclusion criteria were patients diagnosed with primary open angle glaucoma (POAG) who had undergone a trabeculectomy within the study time period. Exclusion criteria included patients with other subtypes of glaucoma (including pseudoexfoliation glaucoma, pigmentary glaucoma, primary angle-closure glaucoma, and uveitic glaucoma), patients undergoing redo-trabeculectomy surgery or trabeculectomy combined with another procedure and unavailability of patient notes.

### Outcome measures

The primary outcome measure was trabeculectomy surgical success, as defined by the World Glaucoma Association guidelines on design and reporting of glaucoma surgical trials [6], and used widely in previous literature [7]. Secondary outcome measures assessed in this study included change in visual acuity, change in visual fields, the requirement for supplemental medical therapy after surgery, surgical complications and the need for any intervention for surgical complications of trabeculectomy surgery.

The baseline IOP for each patient was calculated from a mean of two pre-operative IOP readings. Pachymetry was not a routine measurement at the start of the study period and the IOP was not adjusted for central corneal thickness. Surgical success was defined as IOP ≤ 21 mmHg and > 5 mmHg, or reduced by 20% from baseline. On surgical grounds, failure was defined as IOP > 21 mmHg, ≤ 5 mmHg or not reduced by 20% at two consecutive follow-up visits after 3 months. The time of failure was the second of two consecutive follow-up visits. Failure on functional grounds was deemed to have occurred if the patient required re-operation for elevated IOP, or had loss of light perception vision in the study eye. All eyes that had achieved surgical success with the need of supplemental medical therapy were defined as a qualified success. Eyes that did not require supplemental medical therapy were defined as an absolute success.

### Statistical analysis

Snellen visual acuity measurements were converted to logarithm of the minimal angle of resolution (logMAR) equivalents for the purpose of data analysis. Changes in Humphrey visual field Mean Deviation were plotted for the study patients, with lines of best fit derived by linear regression. Time to failure was assessed using Kaplan-Meier survival analysis. Binary logistic regression analysis was used to calculate odds ratios for failure, qualified success, surgical complications and post-operative interventions with regard to pre-operative IOP, at 3-year follow-up. Significance of these odds ratios were evaluated using Wald *Χ*^2^ tests. A *P* value ≤ 0.05 was considered statistically significant.

## Results

### Sample

A total of 61 patients (80 procedures) were included in the study. Despite repeated attempts, the patient records were unavailable to access for the remaining trabeculectomy cases performed during the study period. The baseline demographic characteristics of the study patients are presented in Table 1. The median time of follow-up was 36 months. All cases had fornix-based trabeculectomy surgery, and mitomycin C was used in 61 of the 80 (76%) procedures. A consultant ophthalmic surgeon performed the surgery in 36 of the 80 (45%) procedures, with the remaining carried out by a trainee or glaucoma fellow.

**Table 1:** **Baseline characteristics of study patients**

	**(** ***n*** **= 80)**
Age at operation (years), mean ± SD	67.3 ± 11.3
Gender, n (%)	
Male	40 (50)
Female	40 (50)
Race, n (%)	
Caucasian	76 (94)
Black	2 (3)
Asian	2 (3)
Diabetes Mellitus, n (%)	6 (8)
Hypertension, n (%)	20 (25)
Glaucoma medications, mean ± SD	2.5 ± 1.0
Lens status, n (%)	
Phakic	68 (85)
Pseudophakic	12 (15)
Snellen visual acuity, LogMAR mean ± SD	0.10 ± 0.15
Pre-operative C/D Ratio, mean ± SD	0.8 ± 0.1
Humphrey visual fields MD, mean ± SD	−9.31 ± 5.75

SD = standard deviation LogMAR = logarithm of the minimal angle of resolution; MD = mean deviation; C/D Ratio = Cup: Disc Ratio.

### Intraocular pressure reduction

**Figure 1:**
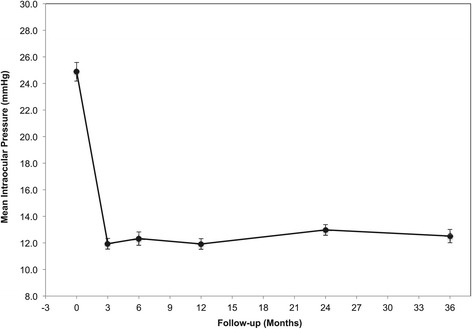
**Distribution of Intraocular Pressure (IOP), at baseline and follow-up.** Baseline IOP for each patient calculated from average of two pre-operative intraocular pressure readings. Mean of these individual readings used as Baseline IOP. Data are presented as mean ± standard error of the mean.

The mean baseline and follow-up IOP measurements for the study patients are shown in Table 2, and illustrated in Figure 1. Thirty eyes included in the study had an intraocular pressure (IOP) consistently less than or equal to 21 mmHg at all clinic visits since diagnosis.

**Table 2:** **Intraocular pressure, visual fields, glaucoma medication** ^*a*^

**Baseline**	
IOP (mmHg)	21.6 ± 6.3
Visual fields MD/dB	−9.3 ± 5.8
Glaucoma medications	2.5 ± 1.0
N (with IOP)	80
3 months	
IOP (mmHg)	11.6 ± 4.0
Visual fields MD/dB	−9.0 ± 8.0
Glaucoma medications	0.2 ± 0.8
N (with IOP)	79
6 months	
IOP (mmHg)	11.9 ± 4.2
Visual fields MD/dB	−9.2 ± 5.9
Glaucoma medications	0.3 ± 0.8
N (with IOP)	79
1 year	
IOP (mmHg)	11.8 ± 3.5
Visual fields MD/dB	−9.7 ± 6.7
Glaucoma medications	0.3 ± 0.9
N (with IOP)	80
2 years	
IOP (mmHg)	12.4 ± 3.8
Visual fields MD/dB	−10.6 ± 6.8
Glaucoma medications	0.5 ± 1.0
N (with IOP)	74
3 years	
IOP (mmHg)	12.3 ± 3.9
Visual fields MD/dB	−11.1 ± 7.0
Glaucoma medications	0.5 ± 1.0
N (with IOP)	53

^*a*^Data are presented as mean ± standard deviation.

POAG = primary open-angle glaucoma; NTG = normal tension glaucoma; the minimal angle of resolution; MD = mean deviation; N = number of patients.

The study patients showed a significant and sustained reduction in IOP from mean baseline after trabeculectomy surgery (*p* < 0.001, paired *t* test). The mean IOP in the study eyes recorded after a 3-year follow-up period showed a 44.2 ± 3.1% (mean ± percentage change standard error [%SE]) reduction from mean baseline IOP.

### Medical therapy

The number of glaucoma medications used by the study patients at baseline and follow-up are shown in Table 2. A significant reduction in use of medical therapy was seen; at 3-year follow-up, the number of glaucoma medications (mean ± standard deviation [SD]) had decreased by 1.8 ± 0.3 (*p* < 0.001, paired *t* test).

### Visual acuity

**Figure 2:**
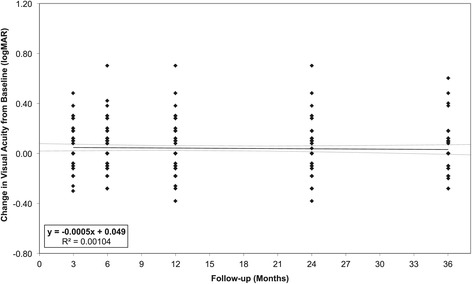
**Change in visual acuity from baseline, at follow-up.** Each point represents a single patient’s change in Snellen Visual Acuity from baseline, with baseline taken from last visit prior to trabeculectomy. A line of best fit obtained from linear regression (maximum likelihood) is shown, with 95% confidence bands. The equation of the line of best fit, along with the R^2^ value, which indicate goodness of fit, is shown in the legend.

The visual acuity (VA) of the study patients, at baseline and follow-up, is shown in Figure 2. There was no significant change in Snellen VA from baseline to 3-year follow-up (*p* = 0.42, paired *t* test).

### Visual fields

**Figure 3:**
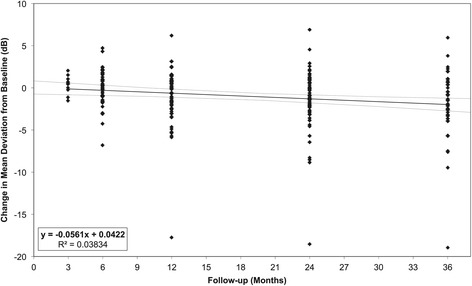
**Change in Humphrey visual field mean deviation from baseline, at follow-up.** Each point represents a single patient’s change in Visual Field Mean Deviation from baseline, with baseline taken from Humphrey Visual Field conducted at last visit prior to trabeculectomy. A line of best fit obtained from linear regression (maximum likelihood) is shown, with 95% confidence bands. The equation of the line of best fit, along with the R^2^ value, which indicate goodness of fit, is shown in the legend.

The Humphrey visual field Mean Deviation (MD), at baseline and follow-up, is shown in Table 2. Changes in Humphrey visual field MD from baseline are shown in Figure 3. There was no significant change in visual field MD from baseline to 3-year follow-up (*p* = 0.11, paired *t* test).

### Trabeculectomy outcomes

**Figure 4:**
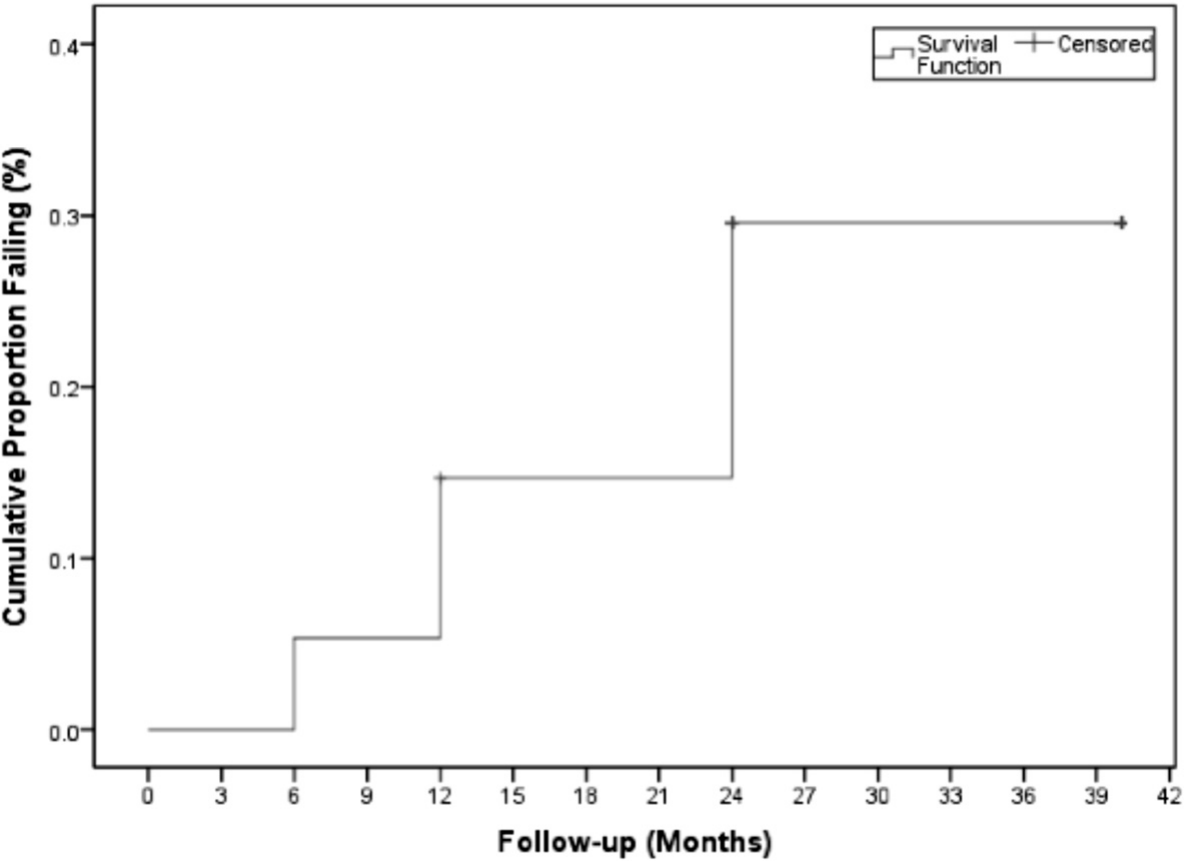
**Kaplan-Meier plot of the probability of failure in the study patients, using 21 mm Hg as the upper limit of success.**

After a 3-year follow-up period, treatment failure had occurred in 10 eyes (13%). 33 of 53 eyes (41%) were classified as an absolute success and 10 eyes (13%) were classified as a qualified success. Kaplan-Meier survival analysis, demonstrating failure rate, is shown in Figure 4. All those eyes that had failed had done so due to inadequate IOP reduction, with no patients requiring reoperation for glaucoma or experiencing loss of light perception.

**Figure 5:**
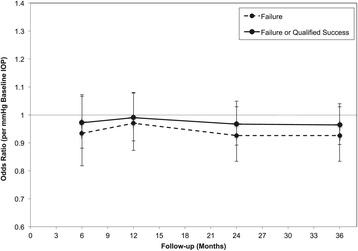
**Odds ratio of failure and of qualified success, at baseline and follow-up.** Data are presented with 95% confidence intervals. (* = *p* < 0.05, ** = *p <* 0.001 according to Wald *Χ* ^2^ test).

The odds ratios of failure of trabeculectomy surgery are illustrated in Figure 5. The odds ratio of trabeculectomy failure at 3 years was 0.93 (95% C.I. 0.83-1.03, *p* = 0.15 Wald *Χ*^2^ test) per mmHg pre-operative IOP. The odds ratio did not differ significantly between 6-month,1-year, 2-year and 3-year follow-ups (*p* > 0.05 at each time-point, Wald *Χ*^2^ test). Failure of trabeculectomy surgery within the period of follow-up showed no association with use of mitomycin C (*p* = 0.369, Fisher’s exact test).

### Post-operative complications

**Figure 6:**
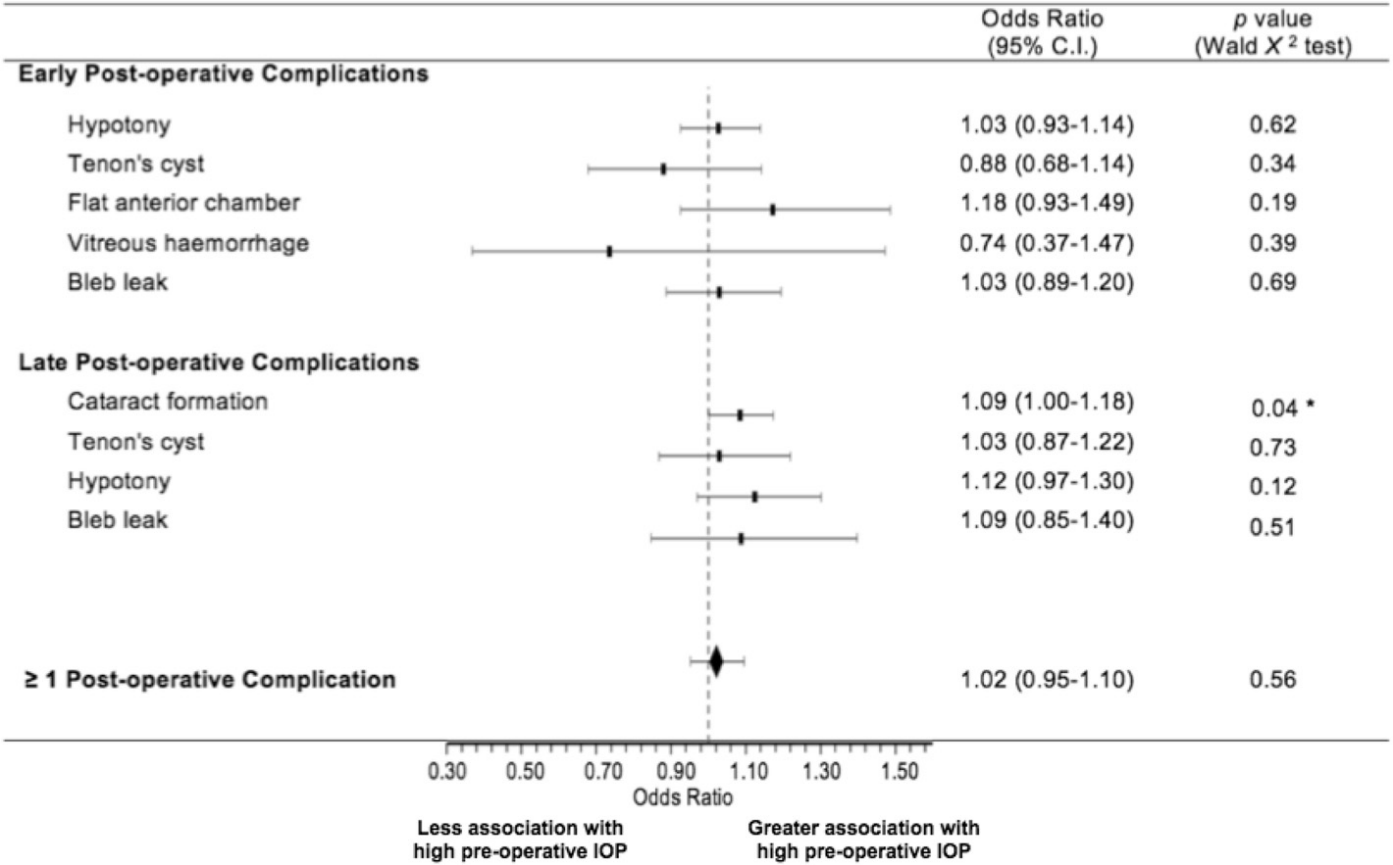
**Forest plot of early and late post-operative complications.** Early complications occurred within 1 month of trabeculectomy surgery, and late complications occurred after this time. Data are presented with 95% confidence intervals. (* = *p* < 0.05, ** = *p <* 0.001 according to Wald *Χ* ^2^ test).

Post-operative complications experienced by the study patients are listed in Table 3. A total of 47 complications in 38 eyes (48%) were reported. Early complications, occurring within the first post-operative month, included hypotony, bleb encystment, flat anterior chamber, vitreous haemorrhage and bleb leak. Late complications, occurring after this time, included cataract formation, hypotony, bleb encystment and bleb leak. The odds ratios for each complication are illustrated using a Forest plot in Figure 6. The odds ratio of having at least one surgical complication was 1.02 per mmHg pre-operative IOP (95% C.I. 0.95-1.10, *p* = 0.55 Wald *Χ*^2^ test). There was a statistically significant odds ratio for cataract formation of 1.09 per mmHg pre-operative IOP (95% C.I. 1.00-1.18, *p* = 0.04 Wald *Χ*^2^ test), and thus increased risk of cataract with a higher pre-operative baseline IOP. No other post-operative complication was significantly affected by pre-operative IOP.

**Table 3:** **Post-operative complications during follow-up**

	**n (%)**
	**(n = 80)**
Early post-operative complications^*a*^	
Hypotony	9 (11)
Tenon’s cyst	3 (4)
Flat anterior chamber	1 (1)
Vitreous haemorrhage	1 (1)
Bleb leak	4 (5)
Late post-operative complications^*b*^	
Cataract formation	22 (28)
Tenon’s cyst	3 (4)
Hypotony	3 (4)
Bleb leak	1 (1)
Total number of patients with post-operative complications^*c*^	38 (48)

^*a*^Onset ≤ 1 month. ^*b*^Onset > 1 month.

^*c*^Some patients had multiple complications.

### Interventions for post-operative complications

**Figure 7:**
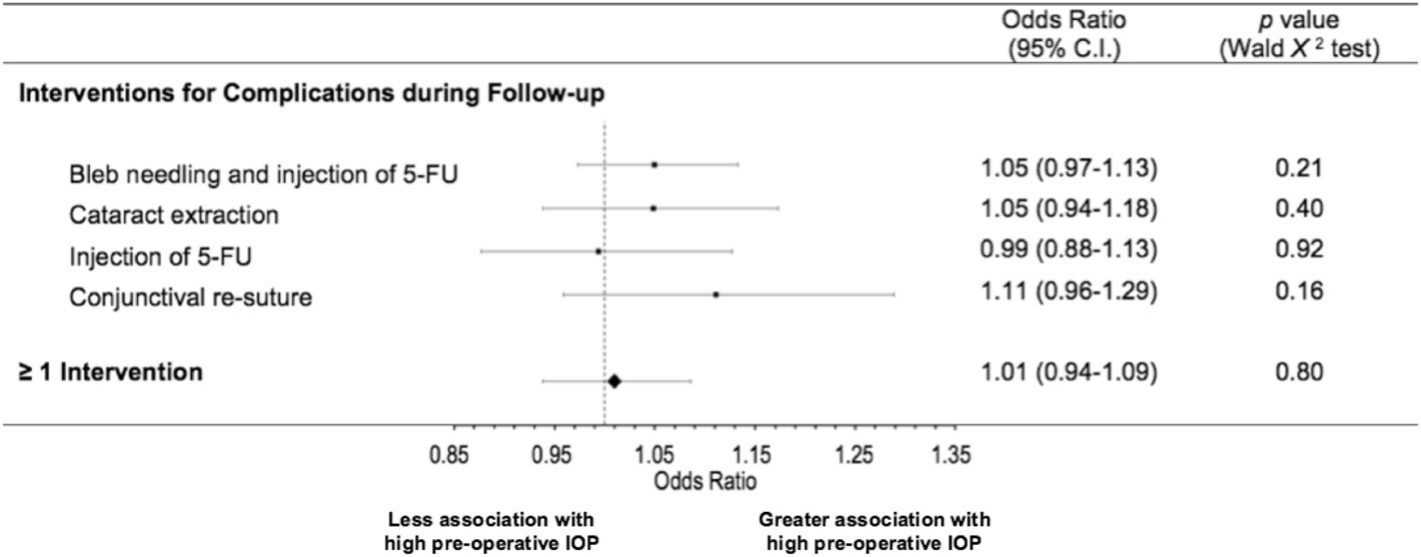
**Forest plot of interventions for complications during follow-up.** Data are presented with 95% confidence intervals. (* = *p* < 0.05, ** = *p <* 0.001 according to Wald *Χ* ^2^ test).

Interventions that were performed for post-operative complications are listed in Table 4, and include bleb needling with 5-FU, cataract extraction, bleb injection with 5-FU and conjunctival re-suturing. A total of 27 patients (34%) required intervention. The odds ratios for each intervention are illustrated using a Forest plot in Figure 7. None of the interventions had an odds ratio significantly different from 1 (*p* > 0.05 Wald *Χ*^2^ test). The incidence of undergoing at least one post-operative intervention showed an odds ratio of 1.01 per mmHg pre-operative IOP (95% C.I. 0.94-1.09, *p* = 0.80 Wald *Χ*^2^ test).

**Table 4:** **Interventions for complications during follow-up**

	**n (%)**
	**(n = 80)**
Bleb needling and injection of 5-FU	22 (28)
Cataract extraction	7 (9)
Injection of 5-FU	7 (9)
Conjunctival re-suture	3 (4)
Total number of patients with interventions for post-operative complications^*a*^	27 (34)

^*a*^Some patients had multiple interventions for post-operative complications.

## Discussion

This retrospective cohort study demonstrated that pre-operative IOP does not predict success of trabeculectomy surgery in medically-uncontrolled POAG patients during the first 3 years of post-operative follow-up. In addition, the incidence of surgical complications and post-operative interventions shows no association with pre-operative IOP.

The absolute IOP at 3-year follow-up was similar in all study eyes, regardless of pre-operative baseline IOP. Further, when patients with a pre-operative IOP <21 mmHg were examined as a separate group to those with preoperative IOP >21 mmHg, the absolute IOP at each post-operative time point was not significantly different between the two groups (*p* > 0.05 at each time-point, Student *t* test).

Despite the relatively small sample size, and the limitations of systematic bias that are present in all retrospective studies, it is encouraging to note that the trabeculectomy outcomes and sample demographics reported by this study mirror those reported by larger studies [4,7,8], suggesting the sample, although limited by its power, is sufficient to draw meaningful conclusions. At 2 years, our cohort had a mean IOP of 12.4 ± 3.8 mmHg, which is identical to that reported by Kirwan *et al.* [4] The success rates seen in our study are comparable with those seen in the Tube Versus Trabeculectomy study [7] which, although a prospective study, used identical definitions for success. After 3 years of follow-up, our cohort of patients showed an overall success rate of 54% (41% absolute success and 13% qualified success). Cataract formation represented the most common surgical complication post-operatively, occurring in 22 eyes (28%), and a similar percentage (20.2%) was reported in the National Survey of Trabeculectomy [8].

Many clinicians divide open-angle glaucoma into primary open angle glaucoma (POAG), where IOP is consistently greater than 21 mmHg, and normal tension glaucoma (NTG), where IOP is consistently less than or equal to this level. This categorical divide has been used by previous studies to stratify outcomes of trabeculectomy [4,5,7]. However, rather than two dichotomous diagnoses, both POAG and NTG are likely to sit on a continuum of open-angle glaucoma. In both sub-types, characteristic features of glaucomatous optic neuropathy and visual field defects are present, but whilst in POAG, these features are caused predominantly by raised IOP, in NTG other IOP-independent factors are thought to be relevant.

The findings of this study are consistent with existing evidence that trabeculectomy is a highly effective treatment for medically-uncontrolled POAG patients, even those with low mean baseline IOP. This study demonstrates that the risk of post-operative complications or the need for post-operative intervention is not heightened if the initial IOP is low. In particular, low pre-operative IOP was not an independent risk factor for hypotony following trabeculectomy surgery.

## Conclusions

In summary, this study demonstrates that pre-operative IOP does not predict success of trabeculectomy surgery, or incidence of surgical complications and post-operative interventions, in POAG patients during the first 3 years of follow-up, a finding of relevance given that a significant proportion of patients do present with low pre-operative IOP (38% in our study, 44% in Kirwan *et al*. [4]). As well as justifying the use of other criteria in addition to IOP in definitions of surgical success [6], this study also highlights the potential bias that may exist in measuring absolute reduction in IOP as an outcome of trabeculectomy surgery, particularly in patients with low pre-operative baseline IOP. A better determinant of functional outcome may indeed be final IOP achieved post-operatively.
